# The Royal College of Surgeons Ward

**Published:** 1915-03-06

**Authors:** W. H. Carden, K. Elmes, J. F. Lyons, M. McMullen, A. Miller

**Affiliations:** Hon. Treasurers.


*To the Editor of* The Hospital.

### 'The Royal College of Surgeons Ward."

*To the Editor of* The Hospital.

Sir,—Your readers are possibly not aware that a very large number of students, past and present, of the Schools of Surgery of the Royal College of Surgeons in Ireland are at present serving with his Majesty's Navy and Expeditionary Forces.

Those at present in the Schools are anxious to help in every possible way they can, and with that object in view they have formed a committee to collect funds to equip a ward in the Dublin Castle Red Cross Hospital, to be called "The Royal College of Surgeons Ward."

The committee would be glad to receive and acknowledge subscriptions from old students and their friends.—We are, yours faithfully,

W. H. Carden, Miss M. McMullen,
K. Elmes, A. Miller,
J. F. Lyons, Hon. Treasurers.

